# Association between fear of falling and self‐care behaviours of older people with hypertension

**DOI:** 10.1002/nop2.1654

**Published:** 2023-02-23

**Authors:** Leila Kouchaki, Ali Darvishpoor Kakhki, Zahra Safavi Bayat, Hafiz T. A. Khan

**Affiliations:** ^1^ Student Research Committee School of Nursing and Midwifery Shahid Beheshti University of Medical Sciences Tehran Iran; ^2^ Department of Medical‐Surgical Nursing School of Nursing and Midwifery Shahid Beheshti University of Medical Sciences Tehran Iran; ^3^ College of Nursing, Midwifery and Healthcare University of West London London UK; ^4^ Present address: The Oxford Institute of Population Ageing The University of Oxford Oxford UK

**Keywords:** accidental falls, aged, fear, hypertension, self‐care

## Abstract

**Aim:**

This study investigated the association between fear of falling and self‐care behaviours of older people with hypertension.

**Design:**

A cross‐sectional study.

**Methods:**

This study was conducted in 2019 on 301 older people with hypertension above the age of 60 years in Tehran, Iran. Data were collected using a demographic questionnaire, the Persian Falls Efficacy Scale‐International, and a hypertension‐related self‐care behaviour questionnaire.

**Results:**

Analyses revealed that gender, educational level and history of falling were significant factors associated with fear of falling; and marital status, educational level and income source were significant factors associated with self‐care behaviours (*p* < 0.05). Partial correlations controlling for education revealed a significant positive correlation showing that high fear of falling is associated with worse health promotion self‐care behaviours and significant inverse correlations with psycho‐emotional, social and daily self‐care behaviours (*p* < 0.05), meaning that high fear of falling is associated with better self‐care for these dimensions.

**Patient or Public Contribution:**

This study involved patients in order to evaluate the validity and reliability of the questionnaires. The study was conducted on older people with hypertension referred to hypertension clinics in hospitals.

## 1 INTRODUCTION

Older adults are the fastest‐growing segment of population globally. The population of adults 60 years or older will rise from 12 to 22% in 2015 and 2050 respectively. These changes are common in some countries such as Iran. The older population in Iran was around 10% in 2015 and will increase to around 33% of the population in 2050 (World Health Organization, 2015).

Although ageing does not necessitate pathological conditions, older adults suffer from chronic illnesses such as hypertension. The rate of hypertension rises with increasing age and nearly 70% of people over 65 years of age have hypertension (Sirkin & Rosner, 2009). In the US and Iran about 63.1 and 67.6% of older adults had hypertension respectively in 2016 (Fryar et al., 2017; Ghaffari et al., 2016).

Hypertension is a main risk factor for heart disease, stroke, kidney failure and diabetes and is a predisposing risk factor for most cardiovascular chronic illnesses (DeWald et al., 2018). Antihypertensive and diuretic medications, polypharmacy and age‐related physiological changes have been associated with an increased risk for falls and consequently fear of falling (FOF) particularly among older adults with hypertension (Kahlaee et al., 2018; Montero‐Odasso et al., 2019). Findings of study by Ozaldemir et al. (2019) showed that hypertensive individuals have higher FOF and decreased functional mobility in comparison with normotensive individuals. This study included participants with a mean age of less than 60 years who had not identified comorbidities. Therefore, it expects FOF to be a more important issue in older adults with hypertension that usually have one or more chronic diseases and more risk factors for falls.

## 2 BACKGROUND

Fear of falling (FOF) has influence on ability of older adults to carry out activities of daily living (Brustio et al., 2018; Hoang et al., 2017; Oh et al., 2017). The restriction of activities caused by fear is also barrier to older adult's presence in society and leads to activity avoidance (Bertera & Bertera, 2008; Mohammadi et al., 2018). Auais et al. (2017) found that FOF was associated negatively with life‐space mobility and social participation of older adults in Canada, Albania, Colombia, and Brazil and is associated with higher levels of loneliness, lower life satisfaction, as well as lower levels of optimism, self‐efficacy, self‐esteem and more perceived stress (Hajek et al., 2018). Mahler and Sarvimaki (2012) found that Finnish older women cope with FOF by managing daily life with a strict daily regime and planning what to do next. Therefore, FOF can influence one's ability to be independent, including the self‐care that is crucial to maintaining health and preventing complications in older adults with hypertension.

Self‐care is the essential component for successful treatment of hypertension (Gholamnejad et al., 2019; Lee & Park, 2017). Self‐care is the personal care that individuals require on a daily basis to regulate their own functioning and development, and is affected by age, developmental stage, environmental conditions and effects of medical care (Orem, 2001). Fundamental principles for self‐care include self‐reliance, autonomy, personal responsibility, self‐efficacy, community participation, community involvement and community empowerment (World Health Organization, 2020). In patients with hypertension, self‐care behaviours encompass smoking cessation, weight management, physical activity and adherence to the diet and medication regimen that is recommended by hypertension guidelines (DeWald et al., 2018).

The association between FOF and ability to engage in self‐care behaviours of older adults with hypertension is not clear, and studies are not asking this question. The aim of our study was to examine the association between FOF and self‐care behaviours of older adults with hypertension.

## 3 THE STUDY

### 3.1 Study design and participants

This cross‐sectional study was conducted in 2019 on older people with hypertension. Participants were referred for hypertension management to the hypertension clinics of eight educational hospitals in Tehran, Iran. Participants were recruited as a convenience sample. The inclusion criteria were age of more than 60 years, ability to speak Persian (national language of Iran), diagnosis of hypertension confirmed by a medical specialist, a hypertension history of more than 6 months, and no history of surgeries which could affect balance, standing and walking during the previous 6 months. A power analysis was performed using the G*Power 3.1.9.7 program to calculate the sample size for the study. A minimum of 260 participants is required with *α* = 0.05, test power = 0.8, and *r* = 0.2 based on the effect size guidelines for ageing studies (Brydges, 2019). Considering dropouts, the number was increased by 15%, for a total of 301 participants.

### 3.2 Data collection

Each participant who met the eligibility criteria was asked to complete self‐report questionnaires. For illiterate participants, the researcher (LK) read the questionnaires for every participant and asked them to give their response.

Data were collected using questionnaires including a demographic and disease questionnaire, the Persian Falls Efficacy Scale‐International (FESI) (Hassankhani et al., 2015), and a hypertension‐related self‐care behaviour questionnaire (Appendix A) developed by the present authors.

Demographics were age, gender, marital status, educational level, income source, living companion, other chronic diseases, body mass index (BMI), duration of hypertension (year) and history of falling in the previous 6 months.

FESI is a 16‐item questionnaire of fall‐related self‐efficacy that was translated into Persian (Hassankhani et al., 2015). This scale measures FOF while doing simple and complex indoor and outdoor physical activities. Its 16 items are scored using a four‐point Likert scale from one (Not at all concerned) to four (Very concerned). Higher values indicate less fall‐related self‐efficacy (and more concern about falling).

The hypertension‐related self‐care behaviours questionnaire was developed based on existing self‐care behaviours questionnaires (Han et al., 2014; Kim et al., 2000; Hemmati Maslak Pak & Hashemlo, 2015), Orem's Self‐Care Deficit Theory (Orem, 2001), and hypertension management guidelines (Fryar et al., 2017; Qaseem et al., 2017). This questionnaire included 49 items (Appendix A) in the five main domains of health promotion (17 items), psycho‐emotional (8 items), social (8 items), spiritual (5 items) and daily (11 items) self‐care behaviours, each rated on a 5‐point scale (Never to Very much). Items 2, 4, 6, 7, 9, 12, 13, 14, 15, 17–22, 24–40, 43, 45, 47 and 49 were reversed so that higher scores indicate poor self‐care behaviours.

The content validity of the Persian version of FESI and the hypertension‐related self‐care behaviour questionnaire was assessed by ten experts in the field of hypertension and ageing. Moreover, the items' clarity and simplicity were assessed by ten older adults with hypertension. Necessary amendments were made based on the experts' and the older adult's comments. The reliability of the Persian version of FESI and the hypertension‐related self‐care behaviour questionnaire was assessed through internal consistency statistics. Cronbach's alpha values were calculated to be 0.9 and 0.8, respectively. Cronbach's alpha for the domains of hypertension‐related self‐care behaviour were 0.9 for physical, 0.9 for psycho‐emotional, 0.9 for social, 0.5 for spiritual and 0.9 for daily self‐care behaviours. Therefore, the reliability of the questionnaires was adequate.

### 3.3 Statistical analysis

We first performed descriptive analyses. Kolmogorov–Smirnov tests confirmed the normality of the FOF and self‐care behaviours variables (*p* < 0.05). Independent‐sample t‐tests, one‐way analysis of variance and post hoc tests, and Pearson's correlation coefficient were used to evaluate the associations between sociodemographic, disease factors and FOF and self‐care behaviours. After that, we developed multiple linear regression models to investigate the effects of sociodemographic, disease characteristics on FOF and self‐care behaviours. Categorical variables were entered into the regression models as dummy/indicator variables in binary form with a reference category for each, for example gender (male = 1 with female = 0 as reference). Finally, partial correlations (partialling out educational level) were calculated between FOF and self‐care behaviours. An alpha level of 0.05 was set to evaluate statistical significance. Data were analysed via the IBM SPSS v25.0 (IBM Corp).

### 3.4 Ethical considerations

Participation in the study was voluntary and anonymous, with the possibility to withdraw at any time or refuse to answer any question without penalty. Participants filled out a written informed consent form and received an orally presented informed consent if they were illiterate. The study protocol was reviewed and approved by the Ethics Committee of REDACTED under the code of PHNM.1395.691.

## 4 RESULTS

The average age of the study participants was 68.6 with a range of 60–86 years. The means of systolic and diastolic blood pressure were 138.9 ± (13.3) and 87.2 ± (10.6) respectively. Other demographic and disease characteristics are shown in Table 1.

**TABLE 1:** Demographic and disease characteristics of participants

Variable	Category	*n*	Percent
Age	60–69	170	56.5
	70–79	104	34.6
	80+	27	9
Gender	Male	136	45.2
	Female	165	54.8
Marital status	Single	22	7.4
	Married	189	62.8
	Widowed	90	29.9
Educational level	Illiterate	105	34.9
	Primary school	76	25.2
	Secondary school	54	17.9
	High school	66	22
Income source	Employed	64	21.3
	Pension	173	57.5
	Charities	64	21.2
Living companion	Spouse	101	33.6
	Children	61	20.2
	Spouse & children	95	31.6
	Alone	44	14.6
Chronic disease	Yes	243	80.7
	No	58	19.3
Body mass index	17–24.9	63	20.9
	25–29.9	181	60.1
	30 +	57	18.9
History of falling	Yes	60	19.9
	No	241	80.1
Duration of hypertension (year)	1–5	131	43.5
	6–10	126	41.9
	11+	44	14.7
Fear of falling	Low (16–32)	206	68.4
	Moderate (33–48)	91	30.2
	High (49–64)	4	1.6
Self‐care behaviours	Low (49–114)	28	9.3
	Moderate (115–179)	270	89.7
	High (180–245)	3	1

Male, and participants with a higher level of education, who were employed, and without a history of falling had higher FOF than those who were female, had lower level of education, had income source from charities, and had a history of falling (Table 2). Significant relationships were not evident between FOF and marital status, and living companion (Table 2). Also, there were no significant correlations between age (*r* = −0.05, *p* = 0.39), duration of hypertension (*r* = −0.01, *p* = 0.93), level of systolic (*r* = −0.09, *p* = 0.11) or diastolic (*r* = 0.01, *p* = 0.89) blood pressure, or BMI (*r* = −0.02, *p* = 0.77) and FOF. When multiple linear regression was conducted, gender, educational level and history of falling remained the significant factors associated with FOF. These variables accounted for 16.1% of the variance in FOF (Table 3).

**TABLE 2:** Relationship between fear of falling, self‐care behaviours, and related factors

Variable	Category	Fear of falling				Self‐care			
		Mean	*F*	*t*	*p*	Mean	*F*	*t*	*p*
Gender	Male	50.15	0.12	0.73	<0.001	150.22	0.91	−0.32	0.747
	Female	46.52				150.83			
Marital status	Single	47.45	2.74		0.066	158.64	4.96		0.008
	Married	48.97				148.61			
	Widowed	46.62				152.67			
Educational level	Illiterate	44.87	11.46		<0.001	155.34	5.62		<0.001
	Primary school	48.50				150.11			
	Secondary school	51.02				146.39			
	High school	50.67				146.86			
Income source	Employed	50.61	3.98		0.020	145.56	4.84		0.009
	Pension	47.65				151.05			
	Charities	47.09				154.22			
Living companion	Spouse	47.61	0.68		0.504	151.26	0.30		0.739
	Children	48.02				150.82			
	Spouse & children	48.83				149.64			
Chronic disease	Yes	48.06	0.49	−0.45	0.653	151.53	0.48	2.14	0.033
	No	48.59				146.47			
History of falling	Yes	45.17	11.02	−3.28	0.005	153.10	5.53	1.36	0.179
	No	48.90				149.92			

*Abbreviations*: *F*, F‐value; *p*, significance level; *t*, *t*‐value.

**TABLE 3:** Analysis of factors associated with fear of falling and self‐care behaviours

Variable	Fear of falling					Self‐care				
	*β*	SE	95% CI		*p*	*β*	SE	95% CI		*p*
			Lower	Upper				Lower	Upper	
Age						0.09	0.16	−0.03	0.21	0.156
Gender										
Female	Ref.									
Male	0.20	0.91	0.09	0.31	0.001					
Marital status										
Married						Ref.				
Single						0.16	0.51	0.05	0.27	0.006
Widowed						0.30	0.10	−0.89	0.49	0.620
Educational level										
Illiterate	Ref.					Ref.				
Primary school	0.13	1.14	0.01	0.25	0.031	−0.07	2.42	−0.20	0.06	0.273
Secondary school	0.26	1.25	0.14	0.38	0.001	−0.13	2.79	−0.26	−0.00	0.046
High school	0.28	1.18	0.16	0.40	0.001	−0.14	2.75	−0.28	0.00	0.054
Income source										
charities	Ref.					Ref.				
Employed	0.01	1.41	−1.28	0.16	0.846	−0.16	2.84	−0.30	−0.02	0.277
Pension	−0.05	1.14	−0.17	0.07	0.432	−0.08	2.34	−0.22	0.06	0.276
Chronic disease										
No						Ref.				
Yes						0.03	2.35	−0.06	0.15	0.511
History of falling										
No	Ref.									
Yes	−0.20	1.08	−0.31	−0.09	0.001					
Duration of hypertension						0.11	0.20	−0.007	0.23	0.067

*Abbreviations*: *β*, standardized coefficients beta; CI, confidence interval; Ref., reference group; SE, standard error.

*Note*: In the fear of falling adjusted *R* ^2^ = 0.161, *R* ^2^ = 0.181 (*F* = 9.24 *p* < 0.001), In the self‐care adjusted *R* ^2^ = 0.160, *R* ^2^ = 0.185 (*F* = 7.36 *p* < 0.001).

The score of self‐care behaviours had significant correlations with participants' age (*r* = 0.2, *p* < 0.001) and duration of hypertension (*r* = 0.2, *p* < 0.001). Married older adults, participants with more education, employment and chronic diseases had on average lower self‐care behaviour scores than those who were unmarried, had lower level of education, had income source from charities, and other chronic diseases (Table 2). Gender, living companion and history of falling had no association with self‐care behaviours (Table 2). Also, there were no significant correlations between the rate of systolic (*r* = 0.07, *p* = 0.22) and diastolic (*r* = 0.07, *p* = 0.20) blood pressure, or BMI (*r* = 0.02, *p* = 0.72) and self‐care behaviours. When multiple linear regression was conducted to identify the factors that were associated with self‐care behaviours, marital status, educational level and income source remained the significant predictors. These variables accounted for 16% of the variance in self‐care behaviours (Table 3).

Partial correlations (partialling out educational level) revealed a significant positive correlation between FOF and the mean score of the health promotion self‐care behaviours, meaning that a high fear of falling is associated with worse health promotion self‐care behaviours. Also, significant inverse correlations were found between FOF and the mean scores of psycho‐emotional, social and daily self‐care behaviours, meaning that high fear of falling is associated with better self‐care for these dimensions (Table 4).

**TABLE 4:** Correlations between fear of falling and self‐care behaviours

	Fear of falling
Self‐Care Behaviours	−0.19**
Health promotion	0.77***
Psycho‐emotional	−0.44***
Social	−0.40***
Spiritual	−0.11
Daily	−0.46***

*Note*: **p < 0.05, ***p < 0.001.

## 5 DISCUSSION

This study aimed to examine the association between FOF and self‐care behaviours among older adults with hypertension.

Among the predictors of FOF, gender, educational level, income source and history of falling were the predictors of FOF. Men experience more FOF than women, which is against the findings of early studies (Hoang et al., 2017; Oh et al., 2017). Probably, age has an important role in these studies as FOF increases in older age (Hoang et al., 2017; Rivasi et al., 2020; Tomita et al., 2018). Our findings showed that participants' average age was less than 70 years and there was no difference between men and women. This age range has been known as ‘young old’ people, and men because of early retirement in Iran are less active than women, which probably causes more experiences of FOF.

Fear of falling (FOF) in older adults increases as the level of education and awareness of FOF increases. Dierking et al. (2016) and Oh et al. (2017) found that more education is a protective factor for FOF in older Mexican Americans and in Korea respectively. A higher level of education is likely an important factor for older adult's awareness of fall risk (Gholamnejad et al., 2019). An increase in awareness would lead to increased information, use of different sources and better social relationships. Therefore, older adults will know more about falling. Older adults without a history of falling had more FOF than older adults with falls. Gardiner et al. (2017) and Hoang et al. (2017) showed that a history of falls can lead to lower independence and more FOF. In addition to high blood pressure that inherently increases concern for falling, high burden of chronic diseases in this study and as showed in other studies is associated with increased FOF (Dierking et al., 2016; Tomita et al., 2018). People who have higher fear of falling take precautions to ensure that they do not fall and it therefore reduces risk of falling.

The predictors of self‐care behaviours were marital status, educational level and income source. Married older adults with higher level of education and their own income had better self‐care behaviours than those who were unmarried, had less education and had income from other sources such as charities. Zhang et al. (2020) showed older patients with hypertension who are single and live alone and have lower education level need to pay more attention to self‐management behaviours such as exercise programs. The findings of Gholamnejad et al. (2019) and Lee and Park (2017) revealed that education level and family support affected self‐care behaviour in older patients with uncontrolled hypertension. Likely education leads to more general health and impetus for doing self‐care behaviours. Education could lead to improvement in job status, social and financial matters, better lifestyle, lower psychological distress and consequently well‐being and access to health services (Gholamnejad et al., 2019). Married older adults have a better family function than unmarried. They receive family support and help from people who are directly concerned about them, particularly their spouse. Therefore, marriage of older adults is along with improve in self‐care behaviours of older adults.

The main finding of this study was that the health promotion aspect of self‐care decreased with increasing FOF. Health promotion self‐care behaviours include specific self‐care behaviours that are necessary to maintain and improve health status of older adults with hypertension. Learning of these behaviours needs capacity to obtain, process and understand necessary health information that is known as health literacy. Clarke et al. (2021) showed fear of recurrence in head and neck cancer survivors is associated with inadequate health literacy. On the other hand, older adults with hypertension must keep adherence to their therapeutic regimen because hypertension is a long‐term disease that needs continuous attention. In addition, 82% of the sample had other chronic diseases that increase care burden and we know that cumulative effects of chronic diseases lead to FOF (Thiamwong & Suwanno, 2017). FOF in the long term will be a barrier to learning and using health promotion self‐care behaviours. Therefore, FOF seems to decrease health promotion self‐care behaviours among older adults with hypertension.

In contrast to the result for health promotion behaviours, significant inverse associations were found between FOF and the dimensions of psycho‐emotional, social and daily self‐care behaviours. Because higher scores on these dimensions indicate poorer self‐care, this means that higher FOF is associated with better functioning in these domains. Although former studies show that FOF is associated with poor activities of daily living (Brustio et al., 2018; Hoang et al., 2017), instrumental activities of daily living (Dierking et al., 2016), depression (Dierking et al., 2016; Hoang et al., 2017; Rivasi et al., 2020), psychological factors (Hajek et al., 2018), and family relationship (Dierking et al., 2016), we found higher FOF associated with a better psycho‐emotional, social and daily self‐care behaviours. Highly probably, older adults with hypertension know about the consequences and complications of their disease, including risk of falling. Gardiner et al. (2017) showed that older adult's feeling ‘at risk of falling’ is a threat to personal identity, independence and social interaction. Dierking et al. (2016) showed frequent friend interaction is a protective factor for FOF. The psycho‐emotional, social and daily self‐care behaviours are universal self‐care behaviours that older adults have enough knowledge and experience about based on lived experiences. They use their experiences to adapt to FOF by better psycho‐emotional, social and daily self‐care behaviours.

Health care workers can design and implement care plans to reduce FOF and improve health promotion self‐care behaviours in older adults with hypertension by considering two categories of modifiable and non‐modifiable factors. For example, they can identify older adults with FOF and design programs for prevention and decrease of falling and FOF at home, hospital, and community. On the other hand, these programs should be based on the living environment of older adults and with regard to the illness and the care received by older adults. Furthermore, encouraging older adults to participate in peer networks and social activities that focus on health promotion self‐care behaviours will improve self‐care behaviours and decrease FOF. Therefore, studies on the effect of interventions to decrease FOF on self‐care behaviours of older adults with hypertension are needed.

### 5.1 Limitations

We had some limitations that may have affected the results our study. Firstly, the study sample was selected from only one of the provinces in Iran and the total sample size was limited. Secondly, we used a newly developed scale for self‐care behaviours of older adults with hypertension that can requires further evaluation of reliability (particularly in the spiritual domain) and validity such as construct validity in future studies. A scale to measure specific self‐care behaviours for older adults with hypertension was not available at the time of the study. Also, self‐reported questionnaires with many items limits interpretation of the results because of problems such as fatigue and precise recall of events and information, particularly in an older population. Lastly, regression models for FOF and self‐care did not explain much of the variation [< 20%], which perhaps can be improved in future studies.

## 6 CONCLUSIONS

This study examined the effects of fear of falling from a multidimensional viewpoint in older adults with hypertension. The results of this study showed that FOF is significantly associated with self‐care behaviours. Although higher FOF goes along with improved psycho‐emotional, social, and daily self‐care behaviours, the health promotion self‐care behaviours that are core for management and control of hypertension in older adults decreased with increasing FOF.

## Funding Statement

This research received no specific grant from any funding agency in the public, commercial, or not‐for‐profit sectors

## 7 ETHICS STATEMENT

Permission to conduct the study was proved by the Ethics Committee of Shahid Beheshti University of Medical Sciences (approval number: PHNM.1395.691). The researcher acquired written informed consent form and received an orally presented informed consent from all participants previous to data collection and their contribution in the study was optional and nameless, with the opportunity of backing away at any time or declining to answer any questions without fine.

## CONFLICT OF INTEREST STATEMENT

The author(s) declare that they have no conflict of interests.

## Data Availability

The data that support the findings of this study are available from the corresponding author upon reasonable request.

## APPENDIX A

### Health promotion self‐care behaviours

1. I use fried foods instead of steamed or steamed foods.
2. I use liquid oils to cook.
3. I take 5 grams (equivalent to one tablespoon) of salt a day.
4. I'm interested in losing weight.
5. I use ready‐made foods like canned, sausage and sausage.
6. I consume four or five medium fruits a day.
7. I consume two glasses of low fat milk and dairy daily.
8. I eat sugar, cake, chocolate, sweets and biscuits.
9. I eat four or five cups of chopped vegetables, salad, or 2.5 or 3.5 cups of boiled vegetables daily.
10. I smoke.
11. I forget my blood pressure medication.
12. I measure my blood pressure at home.
13. I participate in regular physical activities such as walking (30 minutes walking for four to five times a week).
14. I have the ability to take care of myself.
15. I consider the advice of the physician for next visit.
16. I forget the physician's recommendations.
17. I take medicines as order by my physician.

### Psycho‐emotional self‐care behaviours

18 I have enough sleep and rest.
19 I feel energized.
20 I feel comfortable.
21 I feel good about spending my free time with my family and friends.
22 I love myself.
23 I'm unhappy with my life.
24 I feel satisfied to be able to solve my problems.
25 I love others.

### Social self‐care behaviours

26 I avoid anyone who causes discomfort.
27 I avoid any situation that causes discomfort.
28 I'm in touch with my friends or family.
29 Others respect me.
30 I can solve my own problems alone.
31 I can afford the cost of living.
32 I respect others.
33 I can get help if I need help from others.

### Spiritual self‐care behaviours

34 I am able to perform my religious activities, including prayer.
35 I am able to attend religious ceremony.
36 I am grateful for the blessings of God.
37 I am optimistic and hopeful about the future.
38 I help people in need.

### Daily self‐care behaviours

39 I can eat without help.
40 I can take care of my appearance without help (for example, combing my hair).
41 I bath with the help of others.
42 I have trouble getting to the bathroom on time.
43 I can prepare my food myself.
44 I can walk with the help of others.
45 I can go out for shopping.
46 I do my homework with the help of others.
47 I can travel without assistance.
48 I need help from others to take my medications (at the right time and dose).
49 I can wash my personal clothes.
